# *Cannabis sativa* L. Inflorescences from Monoecious Cultivars Grown in Central Italy: An Untargeted Chemical Characterization from Early Flowering to Ripening

**DOI:** 10.3390/molecules25081908

**Published:** 2020-04-20

**Authors:** Cinzia Ingallina, Anatoly P. Sobolev, Simone Circi, Mattia Spano, Caterina Fraschetti, Antonello Filippi, Antonella Di Sotto, Silvia Di Giacomo, Giulia Mazzoccanti, Francesco Gasparrini, Deborah Quaglio, Enio Campiglia, Simone Carradori, Marcello Locatelli, Giuliana Vinci, Mattia Rapa, Salvatore Ciano, Anna Maria Giusti, Bruno Botta, Francesca Ghirga, Donatella Capitani, Luisa Mannina

**Affiliations:** 1 Department of Chemistry and Technology of Drugs, Sapienza University of Rome, Piazzale Aldo Moro 5, 00185 Rome, Italy; cinzia.ingallina@uniroma1.it (C.I.); simone.circi@uniroma1.it (S.C.); mattia.spano@uniroma1.it (M.S.); caterina.fraschetti@uniroma1.it (C.F.); antonello.filippi@uniroma1.it (A.F.); giulia.mazzoccanti@uniroma1.it (G.M.); francesco.gasparrini@uniroma1.it (F.G.); deborah.quaglio@uniroma1.it (D.Q.); bruno.botta@uniroma1.it (B.B.); 2 Institute for Biological Systems, Magnetic Resonance Laboratory “Segre-Capitani”, CNR, Via Salaria Km 29.300, 00015 Monterotondo, Italy; donatella.capitani@cnr.it; 3 Department of Physiology and Pharmacology “V. Ersparmer”, Sapienza University of Rome, P.le Aldo Moro 5, 00185 Rome, Italy; antonella.disotto@uniroma1.it (A.D.S.); silvia.digiacomo@uniroma1.it (S.D.G.); 4 Department of Agriculture and Forest Sciences, University of Tuscia, Via San Camillo de Lellis snc, 01100 Viterbo, Italy; campigli@unitus.it; 5 Department of Pharmacy, University “G. d’Annunzio” of Chieti-Pescara, Via dei Vestini 31, 66100 Chieti, Italy; simone.carradori@unich.it (S.C.); m.locatelli@unich.it (M.L.); 6 Department of Management, Sapienza University of Rome, via del Castro Laurenziano 9, 00161 Rome, Italy; giuliana.vinci@uniroma1.it (G.V.); mattia.rapa@uniroma1.it (M.R.); salvatore.ciano@uniroma1.it (S.C.); 7 Department of Experimental Medicine, Sapienza University of Rome, P.le Aldo Moro 5, 00185 Rome, Italy; annamaria.giusti@uniroma1.it; 8 Center for Life Nano Science@Sapienza, Italian Institute of Technology, Viale Regina Elena 291, 00161 Rome, Italy; francesca.ghirga@iit.it

**Keywords:** *Cannabis sativa* L., monoecious cultivars, inflorescences, cannabinoids, metabolic profile, multimethodological analysis

## Abstract

The chemical composition of the inflorescences from four *Cannabis sativa* L. monoecious cultivars (Ferimon, Uso-31, Felina 32 and Fedora 17), recently introduced in the Lazio Region, was monitored over the season from June to September giving indications on their sensorial, pharmaceutical/nutraceutical proprieties. Both untargeted (NMR) and targeted (GC/MS, UHPLC, HPLC-PDA/FD and spectrophotometry) analyses were carried out to identify and quantify compounds of different classes (sugars, organic acids, amino acids, cannabinoids, terpenoids, phenols, tannins, flavonoids and biogenic amines). All cultivars in each harvesting period showed a THC content below the Italian legal limit, although in general THC content increased over the season. Citric acid, malic acid and glucose showed the highest content in the late flowering period, whereas the content of proline drastically decreased after June in all cultivars. Neophytadiene, nerolidol and chlorogenic acid were quantified only in Felina 32 cultivar, characterized also by a very high content of flavonoids, whereas alloaromadendrene and *trans*-cinnamic acid were detected only in Uso-31 cultivar. Naringenin and naringin were present only in Fedora 17 and Ferimon cultivars, respectively. Moreover, Ferimon had the highest concentration of biogenic amines, especially in July and August. Cadaverine was present in all cultivars but only in September. These results suggest that the chemical composition of *Cannabis sativa* L. inflorescences depends on the cultivar and on the harvesting period. Producers can use this information as a guide to obtain inflorescences with peculiar chemical characteristics according to the specific use.

## 1. Introduction

Industrial hemp, a *Cannabis sativa* L. chemotype with a low content of the psychoactive Δ9-tetrahydrocannabinol (THC), has been traditionally cultivated around the world, especially in Europe, due to its adaptability in a wide range of habitats and its countless properties and uses. Particularly, it has been exploited as a source of textile fibers for the production of dresses, fishing nets, paper, canvas and as a food source. However, during the 70s hemp cultivations gradually have disappeared due to the association with the drug-type *Cannabis sativa* L. rich in THC. After almost 30 years of forgetfulness, the European Union published a Regulation [1] reintroducing the cultivation of some cultivars of *C. sativa* with a THC content lower than 0.2% *w*/*w* for fiber and seed production.

The literature concerning hemp is growing exponentially and covers many different aspects including raw building materials [2], bioenergetic [3], agronomical [4] and pharmaceutical [5,6,7] fields as well as cosmetics and food chemistry [8,9]. Cannabis inflorescences are commonly used to extract cannabinoids for pharmaceutical applications [10] and also to prepare essential oils for nutraceutical products [11]. The higher cannabinoid amount has been found in female inflorescences, when are grown without male plants to prevent pollination and seed formation [12]. Moreover, inflorescence and seed productions were higher in the early flowering genotypes, whereas a high stem yield was achieved through a long vegetative phase of late flowering hemp genotypes [13]. The chemical composition of inflorescences and derived essential oils is determined not only by genetic factors (different cultivars) but also by pedoclimatic conditions and agronomical practices [14,15].

In 2017, the administration of Lazio Region (Central Italy) approved a new regulation [16] regarding the realization of pilot projects aiming at the valorization of local hemp cultivars introduced in regional areas. In this paper, the inflorescences from four monoecious cultivars, Ferimon, Uso-31, Felina 32 and Fedora 17, originated from other countries (France and Ukraine) and only recently introduced in the local areas of Lazio Region in the Central Italy were investigated. Their chemical composition was monitored over the season to give indications on the levels of specific compounds responsible for sensorial and/or pharmaceutical/nutraceutical properties and to assure the low level of THC, as required by law. Considering that cannabinoids have been usually the main targeted compounds for hemp varieties characterization, in order to achieve a more complete phytochemical profile of the cultivar under study, a multi-methodological approach [17,18], including untargeted methodology (NMR) for the metabolic profile and targeted methodologies (UHPLC, GC-MS, HPLC and spectrophotometric analyses) for cannabinoids, terpenoids, phenols, flavonoids, tannins and biogenic amines was applied.

## 2. Results

### 2.1. Chemical Profile of Hemp Inflorescences

The chemical profile of the inflorescences from Ferimon, Felina 32, Uso-31 and Fedora 17 monoecious cultivars, grown in Lazio Region (Central Italy), was investigated through the application of NMR, GC-MS, UHPLC, HPLC-PDA/FD and spectrophotometric methodologies.

NMR is recognized as an untargeted powerful tool [19,20] to give a complete metabolite profile of biological matrixes. Up to now, the NMR based metabolomic investigations regarding *C. sativa* L. have been focused on the study of plant tissues such as trichomes [21], cell suspensions [22] and inflorescences [23]. Only a partial assignment of the inflorescence NMR spectra is available in literature [23,24]. Here, a more complete assignment (Table 1) of the ^1^H-NMR spectra of the hydroalcoholic extracts from *Cannabis sativa* L. inflorescences was reported allowing the identification of different classes of compounds. Six sugars, six organic acids, thirteen amino acids, choline and trigonelline were identified by means of 2D experiments and literature data [18]. The identified compounds turned out to be present in all the investigated samples (both in the four cultivars and in the four harvesting times). Metabolites were quantified using their characteristic ^1^H-NMR signals. Galactose, raffinose, acetic acid, fumaric acid, leucine and tyrosine were not quantified due to a strong signal overlapping.

**Figure 1:**
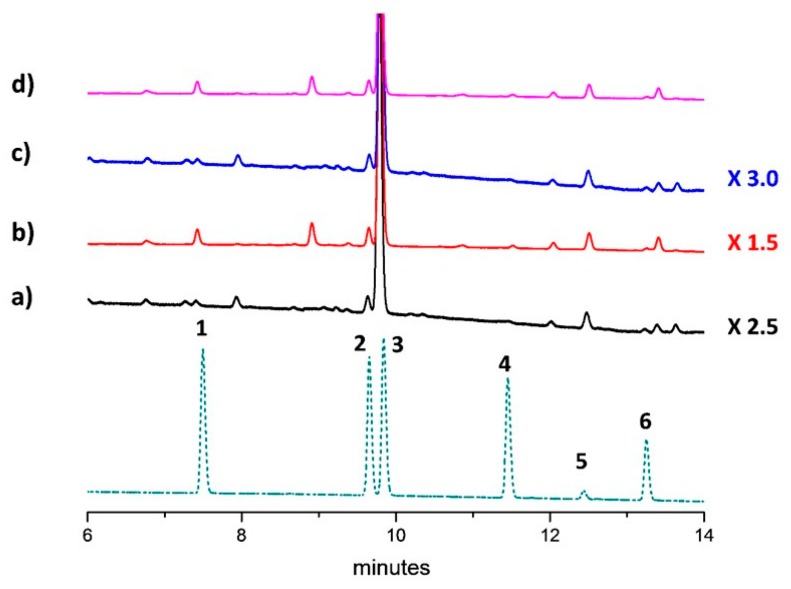

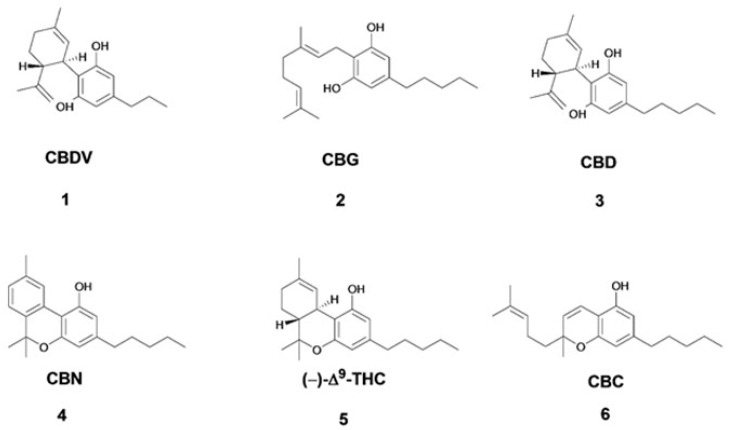
UHPLC chromatograms of inflorescences ethanol extracts from: (**a**) Uso-31 cultivar; (**b**) Felina 32 cultivar; (**c**) Ferimon cultivar; (**d**) Fedora 17 cultivar. For peaks identification, the chromatogram of a six-component cannabinoids standard (**1–6**) mixture has been carried out (bottom).

UHPLC targeted analysis [25] applied to the inflorescences alcoholic extracts provided the cannabinoids profile (Figure 1), including cannabidivarin (CBDV), cannabigerol (CBG), cannabidiol (CBD), cannabinol (CBN), (–)-Δ9- tetrahydrocannabinol (THC) and cannabichromene (CBC) over the season (Table 2).

Fourteen terpenes in Bligh-Dyer organic extracts of samples harvested in June and September were identified by means of GC-MS methodology (Table 3). The compounds identification was achieved by means of mass spectra collected in a commercial database and in free online libraries, confirmed by Kovats index (KI) and standard samples.

The total amount of phenolics, tannins and flavonoids in the organic and hydroalcoholic extracts of June and September samples was measured by spectrophotometric methods (Table 4). Fourteen phenolic compounds were also identified by HPLC-PDA (Table 5).

**Figure 2:**
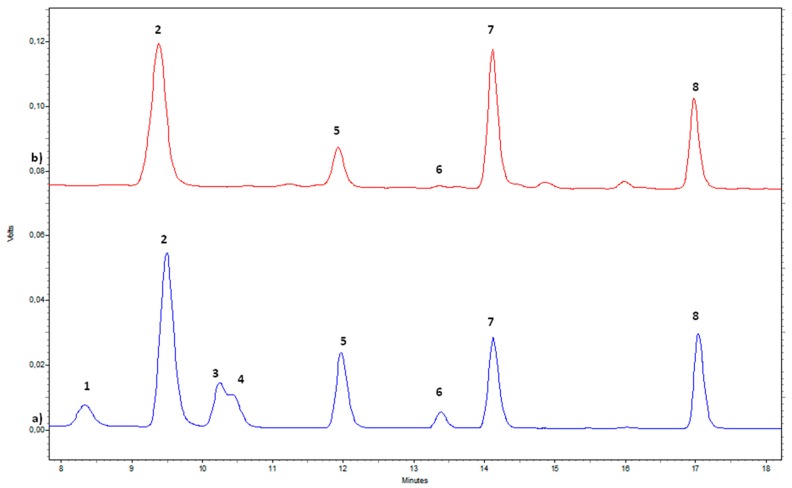
HPLC-FD chromatographic profile of biogenic amines: (**a**) mixture of standard solutions (bottom trace) used for peak identification, (**b**) biogenic amines identified in Ferimon aqueous extract from August (upper trace). Biogenic amines: 1. β-PEA, 2. PUT, 3. CAD, 4. HIS, 5. Internal standard (IS), 6. TYM, 7. SPD, 8. SPM.

The biogenic amines (BAs) presence in the aqueous extracts was verified by means of HPLC-FD (Figure 2). Five out of seven BAs monitored in the samples were present. Putrescine (PUT), tyramine (TYM), spermidine (SPD) and spermine (SPM) were always detected, whereas cadaverine (CAD) was found only in the September samples (Table 6). β-Phenylethylamine (β-PEA) and histamine (HIS) were not detected in the analyzed samples.

### 2.2. Metabolic Profile over the Season of Ferimon, Uso-31, Felina 32 and Fedora 17 Inflorescences

The chemical profile of the inflorescences from each investigated cultivar is discussed separately. Finally, a comparison among cultivars is also reported.

#### 2.2.1. Ferimon Cultivar

**Figure 3:**
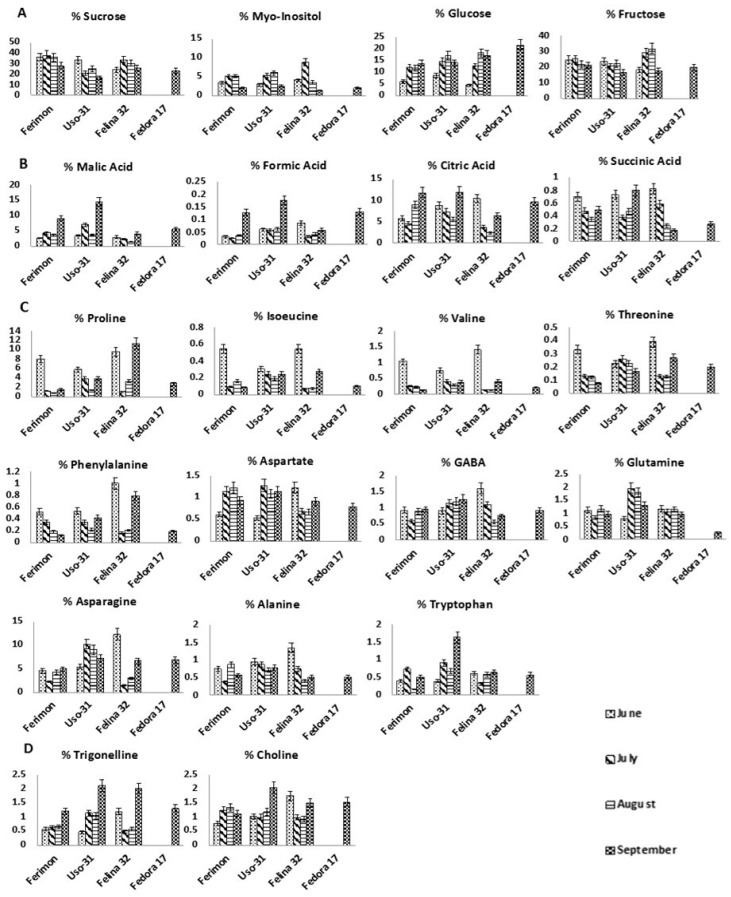
Histograms resulting from the quantitative NMR spectroscopic analysis of some metabolites present in the Bligh-Dyer hydroalcoholic extracts of hemp inflorescences. (**A**). Sugars; (**B**). Organic acids; (**C**). Amino acids; (**D**). Other metabolites.

*Sugars.* Sucrose and fructose showed a similar trend staying constant until August and afterward smoothly decreasing. Glucose content was characterized by an opposite trend, increasing over the season and reaching a maximum in September. Myo-inositol content was found to be the highest in July and August (Figure 3A).

*Organic Acids.* Malic and formic acids contents were constant until August increasing drastically in September, whereas citric acid content was gradually augmented. On the other hand, succinic acid showed the highest content in June decreasing over the season (Figure 3B).

*Free Amino Acids.* Asparagine and proline were the most abundant amino acids. Proline, isoleucine, valine, and threonine showed the highest level in June then drastically decreasing, whereas phenylalanine declined smoothly. Aspartate reached the maximum concentration in July and August, whereas γ-aminobutyrate (GABA), glutamine and asparagine remained quite constant. Alanine and tryptophan were not characterized by specific trends (Figure 3C).

*Miscellaneous compounds.* Trigonelline was constant until August doubling in September, whereas choline reached the maximum concentration in July and August (Figure 3D).

*Cannabinoids.* Among the six cannabinoids identified and quantified, CBD showed the highest concentration that increased over the season (Table 2). The psychotropic compound THC, although grew up during the season, turned out to be always under the limit required by Italian law (max 0.2%). CBG levels increased gradually and remained constant in August and September, whereas in July and August CBN and CBC levels were too low to be detected.

*Terpenoids.* Caryophyllene E, humulene and humulene epoxide were detected in June and September showing an increment over the season, whereas caryophyllene oxide was substantially constant. α-Caryophylladienol, clovanediol and phytol were present only in June.

*Phenolic compounds.* The levels of total phenolics and total tannins were found to be significantly reduced in both the hydroalcoholic and organic extracts from June to September (Table 4). Similarly, flavonoids were reduced about three-fold in the hydroalcoholic extract of September compared to June, whereas in the organic fraction a slight increase occurred (Table 4).

Among the compounds identified by the HPLC-PDA analysis, carvacrol was found in both organic and hydroalcoholic samples. Catechin and rutin were the major compounds in the hydroalcoholic extracts, whereas quercetin, carvacrol and *trans*-ferulic acid (Table 5) were present in lower concentrations. Catechin and rutin decreased from June to September (Table 5). In the organic extracts, catechin, rutin and naringin were present only in June in very low concentrations. Generally, the hydroalcoholic extracts contained the highest levels of phenolic compounds both in June and September.

*Biogenic Amines.* PUT and SPD contents increased from June to August and were reduced in September, whereas SPM increased until July and then decreased. CAD was present only in September samples. Therefore, the total BAs content showed the highest value in July and August (Table 6) suggesting the full seed ripening in these months [26].

#### 2.2.2. Uso-31 Cultivar

Sugars. Sucrose content showed the highest value in June. Fructose remained constant from June to August reaching the minimum in September, whereas glucose content increased over the season. Myo-inositol content was found to be highest in July and August (Figure 3A).

Organic Acids. All organic acids were characterized by the highest content in September. Malic and formic acids were constant until August and increased in September, whereas succinic and citric acids decreased from June to August, then grew up in September (Figure 3B).

Free Amino Acids. Isoleucine, valine, alanine, proline and phenylalanine showed a gradually decrease over the season. Threonine, glutamine and asparagine content reached the maximum level in July then smoothly decreased over the season. Aspartate concentration increased from June to July then remaining constant. Tryptophan content grew up over the season, being three-fold higher in September than in June. GABA content was found to smoothly increase over the season.

Miscellaneous compounds. A drastic increase of both choline and trigonelline content was observed in September.

Cannabinoids. CBD, the main cannabinoid present in the inflorescences (Table 2), showed an increment over the season, achieving in September a level three-time higher than the starting value. THC content increased four-time from June to July, stayed constant from July to August and then increased in September. CBDV increased over the season with the highest rate of growth until August. CBG content decreased from June to July, then returning to the starting value. CBN was detected only in June. CBC was present only in June and July, reaching the highest value in July.

Terpenoids. Caryophyllene E and humulene were detected in June and September showing an increment over the season, whereas caryophyllene oxide and phytol showed an opposite trend. Alloaromadendrene, γ-muurolene, β-selinene α-selinene, humulene epoxide, α-caryophylladienol and clovanediol were present only in September.

Phenolic compounds. Total phenolics, tannins and flavonoids increased from June to September in the organic extracts, whereas in the hydroalcoholic extracts total phenolics and flavonoids showed an opposite trend (Table 4).

Carvacrol amount was reduced from June to September in hydroalcoholic extracts. Moreover, the hydroalcoholic extracts contained rutin and quercetin which were slightly decreased in September with respect to June. The hydroalcoholic sample of June also contained of *t*-cinnamic and *o*-coumaric acids, while that of September contained a relatively high catechin level and a low amount of 3-OH-4-MeO-benzaldehyde (Table 5).

Biogenic Amines. PUT and TYM contents increased from June to August and decreased in September, whereas SPM ad SPD increased until July then decreasing. Therefore, the total BAs content showed the highest value in July and August (Table 6) suggesting the full seed ripening in these months, whereas in September drastically decreased up to 75% (Table 6). As previously observed for Ferimon, CAD was present just in the September samples.

#### 2.2.3. Felina 32 Cultivar

*Sugars.* Sucrose and fructose concentrations showed the highest value in July, then decreasing until September. Glucose content was characterized by an increment over the season. Myo-inositol reached the maximum value in July, but drastically decreased until September.

*Organic Acids.* Formic, citric and succinic acids showed the highest content in June. Malic acid concentration decreased until August and rapidly increased in September.

*Free Amino Acids.* Isoleucine, threonine, phenylalanine, valine, aspartate, asparagine and proline showed a similar trend: their content was the highest in June, drastically dropped down in July and August and then increased in September. GABA and alanine content showed the highest value in June. Tryptophan and glutamine remained quite constant over the season (Figure 3C).

*Miscellaneous compounds.* Choline and trigonelline content was high in June, drastically dropped down in July and August, then increased in September (Figure 3D).

*Cannabinoids.* The cannabinoids content showed some interesting trends (Table 2). Three out of six cannabinoids, CBDV, CBG and CBD reached the highest concentration in August, and then decreased (drastically in the case of CBDV) in September. Interestingly, both CBG and CBDV values were characterized by 10-fold increase from June to August, and both concentrations were found to be lower in September. THC content increased slightly in July and stayed quite constant over the rest of the season. CBN was found only in August and September, whereas CBC was detected only in June and August.

Terpenoids. Caryophyllene E, *trans*-α-bergamotene, humulene, β-selinene, α-selinene, caryophyllene oxide and humulene epoxide grew up from June to September. Nerolidol, neophytadiene and phytol were present only in June, whereas α-caryophylladienol was present only in September.

Phenolic compounds. Felina inflorescences resulted to contain higher amounts of total phenolics and tannins in September with respect to June in both organic and hydroalcoholic extracts (Table 4). Regarding flavonoids, despite a 1.4 increase in the organic extract of September compared to June, the amount in the hydroalcoholic samples showed an opposite trend (Table 4).

Considering the organic extract, carvacrol was the main component that, increased four times from June to September, whereas catechin, *o*-coumaric and *trans*-ferulic acids were present only in September. Rutin was present only in June in the organic extracts. In the hydroalcoholic extracts, the level of catechin, *o*-coumaric acid, quercetin, rutin and *trans*-ferulic acid increased from June to September. It is noteworthy that catechin increased by about 79-fold over the season. Syringic acid, 3-OH-benzoic acid, and 3-OH-4-MeO-benzaldehyde were observed only in September, whereas chlorogenic acid was present only in June.

Biogenic Amines. SPM content increased from June to August and decreased in September, whereas SPD decreased from June to August and slightly increased in September. TYM and PUT showed their highest content in September. Again, CAD was present only in the September sample (Table 6).

#### 2.2.4. Fedora 17 Cultivar

Since Fedora 17 cultivar was collected only in September, it was not possible to follow a metabolite trend over the season.

The same metabolites (sugars, organic acids, amino acids, choline and trigonelline) identified and quantified by NMR spectroscopy in the other cultivars were present in Fedora 17 samples (Table 1 and Figure 3).

Among cannabinoids, only CBDV, CBG, CBD and THC were detected, whereas the following terpenoids were observed: caryophyllene E, humulene, humulene epoxide, β-selinene, α-selinene and caryophyllene oxide.

Phenolics, tannins and flavonoids showed higher amounts in the hydroalcoholic extract with respect to the organic one (Table 4). Among phenolic compounds, carvacrol, rutin and naringenin were found in the organic extract. In the hydroalcoholic extract, catechin and rutin were the most abundant phenolic compounds, whereas lower levels of coumaric acids, carvacrol, quercetin 3-OH-4-MeO-benzaldehyde and phenolic acids, including *t*-ferulic, *t*-cinnamic, chlorogenic and syringic acids were detected (Table 5). At last, PUT, CAD, TYM, SPD and SPM were identified and quantified (Table 6).

## 3. Discussion

The chemical composition of Ferimon, Uso-31, Felina 32 and Fedora 17 cultivars showed common features but also important differences. Regarding the cannabinoids content, relevant to pharmaceutical-nutraceutical *C. sativa* properties it is important to underline that CBD, the most abundant cannabinoid in all cultivars, increased over the season, showing the highest content in Fedora 17 and Felina 32 cultivars at the end of the flowering period. This tendency is in agreement with literature data where it has been observed that THC and CBD content increased with growing degree days [27]. It is important to highlight that all cultivars in each harvesting period showed a THC content below Italian legal limit, although THC content generally increased over the season. This is due to the fact that the analyzed cultivars are CBD-type plants characterized by low levels of THC. Ferimon cultivar showed the lowest THC level with a maximum content in September (0.041%). In a previous HPLC study, the CBD content of ethanolic extracts from Felina 32 and Fedora 17 cultivars has been reported [28]: the harvesting period as well as the geographical area have been not specified making the comparison not perfectly reliable. However, the CBD content reported for Fedora 17 cultivar has been very close to that found in the samples here investigated, whereas Felina 32 cultivar has shown a CBD content higher with respect to that here reported. In another HPLC study [29], the methanol/chloroform extracts of two Futura 75 cultivars collected in August 2017 (geographical areas not reported) have shown a CBD content close to that observed in Felina 32 in August whereas the THC content turned out to be higher than that of the cultivars analyzed in this work.

Sensorial properties of *Cannabis sativa* L. products such as infusions, flavored beer, etc. depend on the content of sugars, organic acids and various secondary metabolites. The highest amount of glucose was observed in the last harvesting periods, whereas sucrose and fructose generally showed an opposite trend. Fedora 17 showed the highest glucose content. Citric and malic acids were the most abundant acids in all the cultivars showing generally an increase in September.

Each cultivar showed a peculiar terpenoidic profile: caryophyllene E, caryophyllene oxide and humulene were always present, but other compounds were observed only in some cultivars or in some periods. For instance, neophytadiene was present only in Felina 32 cultivar in June, whereas alloaromadendrene only in Uso-31 cultivar in September.

Beyond the interest in major cannabinoids, recent researches focused on the presence of various non-cannabinoid metabolites such as polyphenols and benzoic acid derivatives, whose pharmacological and industrial applications could enlarge the potentialities of this plant [30].

Considering the levels of the total phenolics, tannins and flavonoids in September, Ferimon and Felina 32 were the cultivars that showed generally the lowest and the highest level of these compounds, respectively, in all extracts. The only exception was the content of total flavonoids in hydroalcoholic extracts found to be the highest for Fedora 17 instead of Felina 32. As regards June samples, the highest content of the total phenolics, tannins and flavonoids was observed for Uso-31 in the hydroalcoholic extracts, and for Felina 32 in the organic ones.

These data agree with literature, being the phenolic composition in hemp inflorescences reported to be widely variable, due to several factor, among which hemp genotype and harvesting period [31]. The amount of phenolic compounds detected by the HPLC-PDA procedure was higher in the hydroalcoholic extracts for all the selected cultivars, whereas the organic phase was characterized by limited quantities of these secondary metabolites. Catechin, rutin, quercetin and carvacrol were found to be present in almost all hydroalcoholic extracts, with catechin and rutin being the main compounds in September inflorescences of Felina 32. Some phenolic compounds were detected only in specific cultivars: chlorogenic acid in Felina 32, *p*-coumaric acid and naringenin in Fedora 17, *trans*-cinnamic acid in Uso-31 and naringin in Ferimon.

It is important to note that naringenin, naringin, catechin and epicatechin have been found as the most abundant components in another monoecious Futura 75 cultivar recently analyzed with the same chromatographic method [32]. Moreover, differences in phenolic composition have been observed in the case of inflorescences dried extracts from other cultivars such as Futura 75, Kc virtus, Carmagnola Cs and Villanova [33]. Particularly, Futura 75 has been found to be enriched in rutin, whereas Kc virtus has shown lower levels of rutin, catechin and benzoic acid; conversely, phenolic acids (i.e., gallic acid, syringic acid) were found to be ubiquitarian among the cultivars, although with different profiles and amount.

BAs presence in vegetable samples is usually related to the presence of seeds [34,35]. Therefore, their levels in *C. sativa* samples can be correlated to seed presence in the inflorescences [36]. The highest content of total BAs was found in Ferimon cultivar during July and August harvesting periods, mainly due to the high levels of PUT, whereas the lowest content of total BAs was found in Uso-31 cultivar in September. CAD was present only in September at low concentration in all cultivars. However, the levels of BAs in the investigated cultivars were comparable to those detected in other plants with high percentage of protein, as beans [37].

## 4. Materials and Methods

### 4.1. Chemicals and Solvents

Deuterated water (D_2_O) 99.97 atom% of deuterium and 3-(trimethylsilyl)-propionic-2,2,3,3-*d*_4_ acid sodium salt (TSP) were purchased from Euriso-Top (Saclay, France). HPLC-PDA chemical standards, *n*-hexadecane, HIS, SPM, SPD, PUT, (β-PEA), CAD, TYM and 1,7-diaminoheptane were purchased from Sigma-Aldrich (Milan, Italy). Methanol (HPLC-grade), chloroform (HPLC-grade), ethanol (analytical-grade), perchloric acid (70%), acetone (analytical-grade), acetonitrile (HPLC-grade) were obtained from Carlo Erba Reagenti (Milan, Italy). Double-distilled water was obtained using a Millipore Milli-Q Plus water treatment system (Millipore Bedford Corp., Bedford, MA, USA). Sodium carbonate (Na_2_CO_3_; 99.999% purity), Folin-Ciocalteu’s phenol reagent, tannic acid (Ph. Eur. purity) and aluminium chloride hexahydrate (AlCl_3_ × 6 H_2_O; Ph. Eur. purity) were purchased from Merck (Darmstadt, Germany).

Cannabinoids reference standards in methanol CBDV (1 mg/mL), CBG (1 mg/mL), CBD (1 mg/mL), CBN (1 mg/mL), (–)-Δ^9^-THC (0.1 mg/mL) and CBC (1 mg/mL) with purity ≥99%, were purchased from Cerilliant Corporation (Round Rock, TX, USA). For mobile phase, gradient grade water (H_2_O) and acetonitrile (ACN) were purchased from Sigma Aldrich (St. Louis, MO, USA) as well as trifluoroacetic acid (TFA) and analytical grade ethanol used for the extraction procedure. All solvents were further filtered on a 0.2 μm filter.

### 4.2. Hemp Plant Material

The fresh flowering aerial parts from Ferimon, Felina 32, Uso-31 and Fedora 17 monoecious cultivars of *Cannabis sativa* L., belonging to a CBD-rich chemotype [38], were provided by “Canapa Live” cultural association. Ferimon, Felina 32 and Fedora 17 were originated from France, whereas Uso-31 from Ukraine and they are classified as cultivars of different earliness: Ferimon is medium maturing, Uso-31 is early maturing, Felina 32 is medium-late maturing and Fedora 17 is medium-early maturing. The plants were cultivated in experimental fields located in the North Lazio area (Rome, Italy) characterized by a xerofluent soil with a low content of nutrients and organic matter.

The climate of the site is typically Mediterranean characterized by a hot and dry summer with maximum temperatures in July and a mild and wet winter with minimum temperatures in February. The total annual rainfall is approximately 750 mm concentrated mainly in the period October-April.

The hemp cultivars were arranged in the field in a randomized block design with three replications, where the plot size was 100 m^2^ (10 × 10 m). The week before hemp sowing, the experimental fields were fertilized with 100 kg ha^−1^ di P_2_O_5_ as triple superphosphate, afterwards the soil was plowed in and harrowed twice for seedbed preparation.

In the first week of April 2016, the selected hemp cultivars were sown in open field at a seed rate of 6 seeds m^−2^, planting the seeds in rows at 100 cm interrow spacing. One week after the fully emergence, the hemp seedlings were thinned manually at a distance of 50 cm from one another in order to reach the target density of 2 plants m^−2^. Drip irrigation tape was applied on the soil surface on each hemp row in order to supply water and nitrogen fertilizer. Nitrogen fertilization was applied at a ratio of 100 kg ha^−1^ by fertigation, while the amount of irrigation water reintegrated the 90% of water lost through evapotranspiration estimated by an evaporimeter and adjusted by the crop coefficients during the hemp cultivation period.

Hemp inflorescences of Ferimon, Felina 32, and Uso-31 were harvested at four stages corresponding to the reproductive hemp period from early flowering to ripening: June 2016, July 2016, August 2016 and September 2016. Fedora 17 was collected only in September 2016. The inflorescence sampling was carried out following a systematic pattern: 30 plants of each cultivar were collected in the central part of the cultivation area, cutting the upper part (30 cm) of the stem [13]. The inflorescences were then combined to constitute one sample representative of the field at each harvesting time, suitable for the chemical analysis. After harvesting, the fresh plant material was immediately frozen and stored at −80 °C.

### 4.3. Sample Preparation for NMR, GC/MS, Spectrophotometric and HPLC-PDA Analyses

The crop flowering aerial parts were powdered under liquid N_2_ and subjected to the Bligh-Dyer extraction [39]. 3 mL of a mixture of methanol/chloroform (2:1 *v*/*v*), 1 mL of chloroform and 1.2 mL of bidistilled water were sequentially added to 1 g of the powdered sample and the obtained emulsion was preserved at 4 °C for 40 min. The sample was then centrifuged (4200× *g* for 15 min at 4 °C). Hydroalcoholic and organic phases were carefully separated. The pellets were re-extracted using half of the solvent volumes, in the same conditions described above. Both extracts were dried under a N_2_ flow at room temperature and stored at −20 °C until analysis.

### 4.4. Metabolic Profile by NMR Analysis

The dried Bligh-Dyer hydroalcoholic extract of each sample was solubilized in 0.75 mL 400 mM phosphate buffer/D_2_O, containing TSP 1 mM as internal standard and then transferred into a 5 mm NMR tube. NMR spectra of all hydroalcoholic extracts were recorded at 28 °C on an AVANCE 600 spectrometer (Bruker, Milan, Italy) operating at the proton frequency of 600.13 MHz and equipped with a Bruker multinuclear z-gradient 5 mm probe head. ^1^H spectra were referenced to methyl group signals of TSP (δ = 0.00 ppm) in D_2_O. ^1^ H spectra of hydroalcoholic extracts were acquired with 256 transients with a recycle delay of 5 s. The residual HDO signal was suppressed using a pre-saturation. The experiment was carried out by using 45° pulse of 7.50 μs and 32K data points. The two-dimensional (2D) NMR experiments, such as ^1^H-^1^H TOCSY, ^1^H-^13^C HSQC and ^1^H-^13^C HMBC, were carried out under the same experimental conditions previously reported [40]. In order to quantify the metabolites, the integrals of the corresponding selected resonances in ^1^ H-NMR spectra were measured (Table 1) with respect to the standard TSP (1 mM) allowing the molar concentration and the corresponding weight to be calculated. The content (in %) of each metabolite was calculated as ratio of its weight to the total weight of all quantified metabolites. In order to evaluate the repeatability of the protocol, the complete procedure from the extraction to NMR measurement was repeated three times.

### 4.5. Cannabinoids Contents by UHPLC Analysis

Calibration standards solutions in methanol were prepared daily for each analytical batch containing CBDV (**1**), CBG (**2**), CBD (**3**), CBN (**4**), (–)-Δ^9^-THC (**5**) and CBC (**6**) at concentrations: 10, 5, 2, 1 ng/mL for (–)-Δ^9^-THC and 50, 25, 13, 7, 5, ng/mL for the others. The powdered plant material (500 mg) was heated up to 130 °C for 2 h into a glass test tube. Afterwards, the decarboxylated plant material was extracted with analytical grade ethanol (20 mL) in an ultrasound bath for 30 min. The extract was filtered through a 0.45 μm PTFE membrane and finally analyzed.

Analyses were performed on a Shimadzu Nexera ultra high-performance liquid chromatography (UHPLC) system (Shimadzu, Milan, Italy). The Shimadzu Nexera UHPLC was operated using a CBM-20A controller, a SIL-30AC autosampler, four LC-30AD dual-plunger parallel-flow pumps, DGU-20A5 vacuum degasser and a photo diode array detector SPD-M20A (equipped with a semi-micro flow cell of 2.5 μL). The system was controlled by LabSolution software (Shimadzu).

All separations were achieved by using the Titan™ C18 column packed with 1.9 μm fully porous particles (FPP) of narrow particle size distribution. The mobile phase consisted of water (A) and ACN (B), both containing 0.1% TFA. The elution gradient was set as follows: 50% B (0 min), 50% B (1 min), 100% B (16 min), 100% B (20 min), 50% B (21 min) and 50% B (30 min). The flow rate was 0.5 mL/min. The column oven was set at 30 °C. The PDA detector parameters were: sampling rate 100 Hz, wavelength 214 nm. A volume of 1 μL was injected. For reasons of fairness, in crude plant ethanol extracts, it will be referred to THC instead of (–)-Δ^9^-THC, because the two enantiomers of Δ^9^-THC cannot be distinguished by the method used in this work, as instead described in a recent work [25].

Therefore, the proposed method was finally used for qualitative and quantitative analysis of the major cannabinoids present in *Cannabis* material. No complex pre-treatment of sample is necessary before analysis and the ethanol extract can be immediately analyzed. Only a simple filtration step was required to protect the UHPLC column.

Each standard solution was used to construct a calibration curve. Linearity was evaluated by plotting the peak area versus injected concentration. Regression lines were calculated using the least squares method, and linearity was expressed by the R^2^-value. A good linearity was obtained in the range studied for each analyte. With the exception of CBC, the average R^2^-value -value obtained was higher than 0.998 in all cases, indicating a good linearity in the proposed range. The R^2^-value obtained for CBC (0.996) was slightly lower, but still very well acceptable. The obtained calibration curves were subsequently used to determine concentration of cannabinoids in all further experiments. Cannabinoid concentrations are finally shown as % (*w*/*w*) ± SD content of *Cannabis* dry weight (Table 2). Five replications were made for each sample.

### 4.6. Terpenoids Content by Gas Chromatography/Mass Spectrometry (GC/MS)

Bligh-Dyer organic fractions were analyzed by using an Agilent Technologies 6850 gas chromatograph coupled with an Agilent Technologies 5975 mass spectrometer, equipped with HP-5MS capillary column (5% Phenyl 95% Methylpolysiloxane, 30 m × 0.25 mm i.d., film thickness 0.25 µm; Hewlett-Packard, city, CA, USA). GC parameters were adjusted as follows: injector temperature 250 °C, flow rate of the helium carrier gas (99.995% purity) 1.0 mL/min. The oven temperature was set at 40 °C (5 min), then raised to 200 °C at 5 °C/min and maintained at this temperature for 60 min. MS parameters were set as follows: energy of electron ionization 70 eV, solvent delay 6 min, source temperature 230 °C, quadrupole temperature 150 °C, and mass scan carried out over the 50–350 m/z range.

The eluted compounds were identified by matching the relative mass spectra with those available from both commercial database (FFNSC 3) and online libraries (NIST 11, Flavor2). Kovats index (KI) was used as a second parameter to confirm the analytes identification: KI has been measured by using a mixture of *n*-alkanes (C_8_–C_24_) in the same analytic conditions and then compared with values reported in literature and in the FFNSC 3 database. The identity of several compounds has been also confirmed through the injection of standard samples available from commercial sources. The relative abundances of each component were obtained by integrating the GC/MS peak areas calibrated by correction factors relied on an internal standard (*n*-hexadecane).

### 4.7. Total Phenolics, Tannins and Flavonoids by Spectrophotometric Methods

The total content of phenolics, tannins and flavonoids in the Bligh-Dyer extracts was determined according to previously standardized spectrophotometric methods [32]. To perform the analysis, the organic and hydroalcoholic dry extracts were dissolved in 100% and 50% *v/v* EtOH, respectively. For the total phenolics, each sample (20 μL) was mixed with the Folin-Ciocalteu’s reagent (100 μL; 10% *v*/*v*) and incubated for 5 min. Then, a sodium carbonate solution (80 μL; 7.5% *w*/*v*) was added, shaken and incubated for 2 h again. The tannin content was evaluated by mixing equal volumes of a polyvinylpyrrolidone (PVP) water solution (100 mg/mL) and the tested sample (1 mg/mL). Tannins bind to PVP forming an insoluble precipitate, so that the supernatant fraction can be collected after centrifugation at 800 g for 10 min. The tannin amount was determined by the difference between the phenolic content in the mixture without PVP and in the supernatant fraction, as measured by the Folin-Ciocalteu’s method. For both phenolics and tannins, the absorbance was measured at 765 nm and the amount was calculated as tannic acid equivalents (TAE). For the total flavonoids, equal volumes of aluminium trichloride (2% *w/v* in methanol) and the tested sample (100 μg/mL) were mixed and incubated for 10 min. The absorbance was measured at 415 nm and the flavonoid content was expressed as quercetin equivalents (QE). Significant differences in the levels of the analyzed chemical classes among the cultivars were evaluated by one-way analysis of variance (one-way ANOVA), followed by Bonferroni’s Multiple Comparison Post Test. Significant differences between the levels in the same cultivar in June and September were analyzed by the t-Student Test. A *p* value < 0.05 was considered significant.

### 4.8. Phenolic Content by HPLC-PDA

The phenolic profile was detected following a validated method applied to previous analyses of *Cannabis sativa* L. essential oils and aqueous flower extracts [32]. All the samples were weighted, solubilized in the mobile phase and directly injected (20 µL). For over range samples, 1:10 dilution factor was applied. Data are described as mean ± standard deviation of three independent measurements. Compounds with values below Limit Of Detection (LOD) or Limit Of Quantification (LOQ) were omitted.

### 4.9. Biogenic Amines (BAs) by HPLC-FD

HIS, SPM, SPD, PUT, β-PEA, CAD, TYM and 1,7-diaminoheptane (IS) were determined according to a previously optimized method [18]. Briefly, 1 g of inflorescence sample previously added with IS (0.5 mL) was extracted twice with 0.6M HClO_4_ (15 + 10 mL), homogenized (3 min), centrifuged (2500× *g* for 10 min) and filtered. The final volume was adjusted to 25 mL with 0.6M HClO_4_. The pre-column derivatization and the analytical determination were carried out as previously reported.

## 5. Conclusions

All the obtained results indicate that each monoecious cultivar has a characteristic chemical profile that changes during the season. Indications of the levels of specific compounds responsible for sensorial and/or pharmaceutical-nutraceutical properties could be useful for the industries which use *Cannabis sativa* L. based products. Further studies could be carried out in order to evaluate a possible pharmaceutical interest and biological activity for specific phytocomplexes of these cultivars.

## Figures and Tables

**Table 1:** Metabolites identified in the 600.13 MHz ^1^ H-NMR spectra of the Bligh-Dyer hydroalcoholic extracts of *Cannabis sativa* L. inflorescences dissolved in 400 mM phosphate buffer/D_2_O containing TSP 1 mM.

Compound	Assignment	^1^H (ppm)	Multiplicity [J(Hz)]	^13^C (ppm)
**Sugars**				
α-d-Fructofuranose	CH-3	4.14		83.1
	CH-5	4.07 *		82.6
β-d-Fructofuranose	CH-3	4.12		76.9
	CH-4	4.12		75.9
	CH-5	3.85		81.7
β-d-Fructopyranose	CH-3	3.81		67.1
	CH-5	4.05 *		66.8
	CH_2_-6,6′	3.71; 4.03		64.4
α-Galactose	CH-1	5.28	d [3.8]	90.2
	CH-2	3.78		
	CH-3	3.83		
	CH-4	3.87		
	CH-5	4.08		
β-Galactose	CH-1	4.60	d [8.0]	97.4
	CH-2	3.51		
	CH-3	3.67		
	CH-4	3.95		
	CH-5	4.05		
	CH-6	3.78		
α-Glucose	CH-1	5.25 *	d [3.8]	93.1
	CH-2	3.56		72.2
	CH-3	3.74		73.8
	CH-4	3.45		70.7
	CH-5	3.84		72.5
	CH_2_-6,6′	3.86; 3.79		60.1
β-Glucose	CH-1	4.66 *	d [8.0]	97.0
	CH-2	3.27		75.2
	CH-3	3.51		76.8
	CH-4	3.43		70.7
	CH-5	3.48		75.1
	CH_2_-6,6′	3.90; 3.74		61.9
Myo-Inositol	CH-2,5	3.56		
	CH-3,6	3.65		
	CH-4	3.30 *		74.2
Sucrose	CH-1 (Glucose)	5.41 *	d [3.8]	93.3
	CH-2	3.57		71.8
	CH-3	3.78		73.6
	CH-4	3.49		70.2
	CH-5	3.85		73.5
	CH_2_-6	3.83		63.5
	CH_2_-1′ (Fructose)	3.69	d [3.3]	60.6
	CH-3′	4.23	d [8.7]	77.5
	CH-4′	4.06	t [8.7]	75.1
	CH-5′	3.9		82.4
	CH_2_-6′	3.82		61.2
Raffinose	CH-1 (Galactose)	5.01	d [3.8]	99.4
	CH-2	3.85		
	CH-3	3.91		
	CH-4	4.03		
	CH-1 (Glucose)	5.44	d [3.9]	
	CH-2	3.59		
	CH-3	3.78		
	CH-5	4.08		
	CH-3 (Fructose)	4.24	d [8.7]	
**Organic acids**				
Acetic acid	CH_3_	1.93	s	24.7
	COOH			180.3
Citric acid	α,γ-CH	2.56 *	d [15.9]	46.2
	α,γ′-CH	2.69		46.2
	β-C			74.2
	1,5-COOH			177.7
	6-COOH			180.2
Formic acid	HCOOH	8.47 *	s	
Fumaric Acid	α,β-CH=CH	6.53	s	
Malic acid	α-CH	4.31 *	dd [9.8; 3.2]	71.4
	β-CH	2.70	dd [15.6; 3.2]	43.9
	β′-CH	2.39	dd [15.6; 9.8]	43.9
Succinic acid	α,β-CH_2_	2.42 *	s	35.2
**Amino acids**				
Alanine	α-CH	3.81		51.6
	β-CH_3_	1.49 *	d [7.3]	17.2
	COOH			174.5
Asparagine	α-CH	4.02		52.3
	β,β′-CH_2_	2.89; 2.97 *		35.8
Aspartate	α-CH	3.91		52.3
	β,β′-CH_2_	2.72; 2.82 *	dd [3.9; 17.4]	37.5
γ-Aminobutyrate	α-CH_2_	2.31 *	t [7.4]	37.2
	β-CH_2_	1.92		24.6
	γ-CH_2_	3.04	t [7.6]	40.2
Glutamine	α-CH	3.78		55.9
	β,β′-CH_2_	2.18	m	27.3
	γ-CH	2.46 *	m	31.8
Isoleucine	α-CH	3.69		
	β-CH	1.98		
	γ-CH_3_	1.02 *	d [7.0]	15.8
	δ-CH_3_	0.94		
Leucine	α-CH	3.77		
	β-CH_2_	1.74		
	γ-CH	1.71		
	δ-CH_3_	0.97		23.1
	δ′-CH_3_	0.96		22.0
Phenylalanine	CH-2,6	7.34		130.5
	CH-4	7.38		128.7
	CH-3,5	7.43 *	m	130.2
Proline	α-CH	4.14		62.4
	γ-CH_2_	2.01 *	m	24.9
Threonine	α-CH	3.62		61.4
	β-CH	4.28		68.1
	γ-CH_3_	1.34 *	d [6.6]	18.9
Tryptophan	CH-4	7.71	d [7.8]	119.6
	CH-7	7.52 *	d [7.8]	113.0
Tyrosine	CH-3,5	7.19		131.7
	CH-2,6	6.90		116.9
Valine	α-CH	3.63		
	β-CH	2.28		30.1
	γ-CH_3_	1.00	d [7.03]	17.8
	γ′-CH_3_	1.05 *	d [7.03]	19.1
**Miscellaneous metabolites**				
Choline	^+^N(CH_3_)_3_	3.21 *	s	54.8
Trigonelline	CH-1	9.11 *	s	
	CH-3,5	8.84		
	CH-4	8.11		

Asterisks (*) indicate signals selected for integration.

**Table 2:** UHPLC cannabinoids concentration in Ferimon, Uso-31, Felina 32 and Fedora 17 cultivars over the season. Results were reported as % (*w*/*w* of dried sample) ± SD (standard deviation), *n* = 5.

Cultivar	Harvesting Period	CBDV	CBG	CBD	CBN	THC	CBC
Ferimon	June	0.0100 ± 0.0004	0.0210 ± 0.0008	0.3800 ± 0.0097	0.0030 ± 0.0001	0.0220 ± 0.0006	0.0400 ± 0.0014
	July	^-^	0.0310 ± 0.0008	0.4420 ± 0.0103	^-^	0.0300 ± 0.0012	^-^
	August	0.0200 ± 0.0008	0.0410 ± 0.0013	0.5010 ± 0.0131	^-^	0.0320 ± 0.0011	^-^
	September	0.0300 ± 0.0007	0.0410 ± 0.0015	0.7010 ± 0.0185	0.0030 ± 0.0001	0.0410 ± 0.0014	0.0500 ± 0.0020
Uso-31	June	0.0030 ± 0.0001	0.0410 ± 0.0009	0.2700 ± 0.0057	0.0040 ± 0.0001	0.0200 ± 0.0004	0.0400 ± 0.0013
	July	0.0080 ± 0.0002	0.0200 ± 0.0007	0.4610 ± 0.0093	^-^	0.0800 ± 0.0021	0.1200 ± 0.0024
	August	0.0210 ± 0.0005	0.0400 ± 0.0009	0.6500 ± 0.0130	^-^	0.0800 ± 0.0020	^-^
	September	0.0320 ± 0.0008	0.0430 ± 0.0010	0.8400 ± 0.0169	^-^	0.0910 ± 0.0021	^-^
Felina 32	June	0.0500 ± 0.0010	0.0300 ± 0.0009	0.8120 ± 0.0171	^-^	0.0600 ± 0.0019	0.0700 ± 0.0023
	July	0.2800 ± 0.0084	0.0610 ± 0.0018	1.1300 ± 0.0285	^-^	0.0830 ± 0.0028	^-^
	August	0.5000 ± 0.0101	0.3100 ± 0.0093	1.4100 ± 0.0284	0.0310 ± 0.0010	0.0800 ± 0.0019	0.0500 ± 0.0018
	September	0.0810 ± 0.0026	0.2200 ± 0.0065	1.1400 ± 0.0295	0.0400 ± 0.0093	0.0730 ± 0.0024	^-^
Fedora 17	September	0.1200 ± 0.0032	0.0410 ± 0.0013	2.0200 ± 0.0405	^-^	0.0700 ± 0.0019	^-^

**Table 3:** GC-MS terpenoids content in Bligh-Dyer organic extracts from inflorescences of Ferimon, Uso-31, Felina 32 and Fedora 17 cultivars harvested in June and September. Results were expressed as [area percentage] mean ± SD (standard deviation), *n* = 3.

Terpenoids	Ferimon		Uso-31		Felina 32		Fedora 17
	June	September	June	September	June	September	September
Caryophyllene E	15.2 ± 0.49	28.0 ± 2.00	6.3 ± 0.48	11.0 ± 0.49	16.4 ± 0.45	25.0 ± 0.49	20.0 ± 0.49
*Trans*-α-Bergamotene	-	-	-	-	1.9 ± 0.05	4.2 ± 0.25	-
Humulene	7.5 ± 0.32	9.0 ± 0.47	1.3 ± 0.04	4.7 ± 0.23	10.9 ± 0.45	16.7 ± 0.50	5.0 ± 0.25
Alloaromadendrene	-	-		3.2 ± 0.14	-	-	-
γ-Muurolene	-	-		1.6 ± 0.46	-	-	-
β-Selinene	-	-	-	3.9 ± 0.14	3.6 ± 0.15	8.3 ± 0.50	5.0 ± 0.23
α-Selinene	-	-	-	3.2 ± 0.15	1.9 ± 0.06	4.2 ± 0.20	5.0 ± 0.23
Nerolidol	-	-	-	-	3.6 ± 0.14	-	-
Caryophyllene oxide	50.0 ± 2.48	49.0 ± 2.52	72.2 ± 3.48	46.4 ± 2.30	10.9 ± 0.47	25.0 ± 1.20	45.0 ± 1.60
Humulene epoxide	10.6 ± 0.49	14.0 ± 0.39	-	11.0 ± 0.45	5.4 ± 0.40	8.3 ± 0.45	10.0 ± 0.35
α-Caryophylladienol	6.1 ± 0.25	-	-	6.3 ± 0.42	-	8.3 ± 0.44	10.0 ± 0.38
Clovanediol	4.5 ± 0.28	-	-	5.5 ± 0.26	-	-	-
Neophytadiene	-	-	-	-	5.4 ± 0.38	-	-
Phytol	6.1 ± 0.26	-	20.2 ± 1.00	3.2 ± 0.13	40 ± 1.90	-	-

**Table 4:** Total polyphenols, tannins and flavonoids content in the hydroalcoholic (HA) and organic (O) Bligh-Dyer extracts obtained from the June and September harvested inflorescences of Ferimon, Uso-31, Felina 32 and Fedora 17 cultivars. Values were expressed as [mg/g of fresh sample] mean ± SD (standard deviation), *n* = 6.

*Cultivar* Harvesting Period	Total Polyphenols		Total Tannins		Total Flavonoids	
	[mg TAE/g]		[mg TAE/g]		[mg QE/g]	
	HA	O	HA	O	HA	O
*Ferimon*						
June	1.75 ± 0.01 ^§,c^	0.95 ± 0.03 ^§,b^	0.90 ± 0.02 ^§,c^	0.32 ± 0.02 ^§,b^	3.02 ± 0.03 ^§,c^	0.47 ± 0.01 ^§,b^
September	0.78 ± 0.06 *^,§,b,c,d^	0.71 ± 0.02	0.39 ± 0.02 *	0.18 ± 0.01 *	1.03 ± 0.03 *	0.67 ± 0.01 *
*Uso-31*						
June	2.11 ± 0.03 ^§,a,c,d^	0.52 ± 0.05	0.89 ± 0.03 ^§,c^	0.25 ± 0.01	4.07 ± 0.03 ^§,a,c^	0.30 ± 0.03
September	1.78 ± 0.02 *	1.11 ± 0.02 *^,§,a^	1.00 ± 0.01 ^§,a^	0.68 ± 0.04 *^,§,a^	2.44 ± 0.01 *^,§,a,c^	0.99 ± 0.01 *^,§,a,d^
*Felina 32*						
June	1.51 ± 0.03	1.69 ± 0.05 ^§,a,b^	0.71 ± 0.01	0.97 ± 0.03 ^§,a,b^	1.63 ± 0.03	6.27 ± 0.05 ^§,a,b^
September	4.00 ± 0.01 *	4.67 ± 0.03 *^,§,a,b,d^	4.00 ± 0.03 *^,§,a,b,d^	2.87 ± 0.03 *^,§,a,b,d^	1.16 ± 0.01 *	8.72 ± 0.05 *^,§,a,b,d^
*Fedora 17*						
September	1.86 ± 0.04 ^§,c^	1.54 ± 0.02 ^§,a,b^	0.94 ± 0.01 ^§,a^	0.58 ± 0.02 ^§,a,b^	3.82 ± 0.02 ^§,a,b,c^	0.57 ± 0.03

TAE, tannic acid equivalents; QE, quercetin equivalents. * *p* < 0.01, significantly different from the level in the same cultivar in June (*t*-Student Test). ^§^ *p* < 0.05, significantly different than the other cultivars in the same harvesting period (one-way ANOVA, followed by Bonferroni’s Multiple Comparison Post Test). ^a^ vs. Ferimon. ^b^ vs. Uso31. ^c^ vs. Felina 32. ^d^ vs. Fedora 17.

**Table 5:** HPLC-PDA phenolic composition of the organic (O) and hydroalcoholic (HA) Bligh-Dyer extracts from Ferimon, Uso-31, Felina 32 and Fedora 17 cultivar inflorescences harvested in June and September. Values were expressed as [μg/mg of fresh sample] mean ± SD (standard deviation), *n* = 3.

Compound	Harvesting	Ferimon		Uso-31		Felina 32		Fedora 17	
		HA	O	HA	O	HA	O	HA	O
Carvacrol	June	0.044 ± 0.004	0.025 ± 0.002	0.123 ± 0.010	0.036 ± 0.003	-	0.031 ± 0.003	-	-
	September	0.018 ± 0.002	0.028 ± 0.002	0.059 ± 0.005	0.050 ± 0.005	0.138 ± 0.015	0.127 ± 0.012	0.062 ± 0.006	0.055 ± 0.004
Catechin	June	0.450 ± 0.044	0.021 ± 0.002	-	-	0.047 ± 0.003	-	-	-
	September	0.194 ± 0.015	-	0.782 ± 0.078	-	3.723 ± 0.357	0.107 ± 0.009	0.657 ± 0.064	-
Rutin	June	0.872 ± 0.094	0.008 ± 0.001	0.666 ± 0.068	-	0.716 ± 0.074	0.026 ± 0.002	-	-
	September	0.436 ± 0.038	-	0.598 ± 0.049	-	3.787 ± 0.280	-	0.660 ± 0.066	0.018 ± 0.002
Quercetin	June	0.047 ± 0.005	0.007 ± 0.001	0.069 ± 0.007	-	0.046 ± 0.004	-	-	-
	September	0.028 ± 0.002	-	0.048 ± 0.005	-	0.125 ± 0.013	-	0.033 ± 0.003	-
Naringenin	June	-	-	-	-	-	-	-	-
	September	-	-	-	-	-	-	-	0.011 ± 0.001
Naringin	June	-	0.007 ± 0.001	-	-	-	-	-	-
	September	-	-	-	-	-	-	-	-
*o*-Coumaric acid	June	-	-	0.469 ± 0.043	-	0.081 ± 0.009	-	-	-
	September	-	-	-	-	0.425 ± 0.038	0.015 ± 0.001	0.055 ± 0.005	-
*p*-Coumaric acid	June	-	-	-	-	-	-	-	-
	September	-	-	-	-	-	-	0.098 ± 0.008	-
Syringic acid	June	-	-	-	-	-	-	-	-
	September	-	-	-	-	0.110 ± 0.010	-	0.020 ± 0.001	-
*trans*-Cinnamic acid	June	-	-	0.038 ± 0.003	-	-	-	-	-
	September	-	-	-	-	-	-	-	-
Chlorogenic acid	June	-	-	-	-	0.320 ± 0.032	-	-	-
	September	-	-	-	-	-	-	-	-
*trans*-Ferulic acid	June	-	-	-	-	0.023 ± 0.001	-	-	-
	September	0.004 ± 0.001	-	-	-	0.092 ± 0.008	0.029 ± 0.003	0.015 ± 0.001	-
3-OH-benzoic acid	June	-	-	-	-	-	-	-	-
	September	-	-	-	-	0.072 ± 0.007	-	0.044 ± 0.004	-
3-OH-4-MeO-benzaldehyde	June	-	-	-	-	-	-	-	-
	September	-	-	0.026 ± 0.002	-	0.565 ± 0.043	-	0.060 ± 0.006	-

**Table 6:** HPLC-FD quantification of biogenic amines in the aqueous extracts of Ferimon, Uso-31, Felina 32 and Fedora 17 cultivars over the season. Results were expressed as [mg/Kg of fresh sample] mean ± SD (standard deviation), *n* = 3.

Cultivar Harvesting Period	PUT	CAD	TYM	SPD	SPM	Total BAs
*Ferimon*						
June	27.97 ± 4.34	-	-	35.53 ± 4.08	14.44 ± 2.01	77.94 ± 9.83
July	81.33 ± 7.95	-	13.20 ± 1.91	55.48 ± 1.00	59.68 ± 5.19	209.69 ± 15.84
August	102.72 ± 5.92	-	2.19 ± 1.41	65.71 ± 5.48	40.93 ± 2.36	211.56 ± 14.86
September	12.79 ± 1.27	5.54 ± 0.05	9.10 ± 0.87	38.65 ± 0.94	27.93 ± 1.29	94.00 ± 1.14
***Uso-31***						
June	55.93 ± 4.27	-	6.35 ± 3.16	41.53 ± 0.41	15.13 ± 0.86	118.94 ± 8.06
July	61.64 ± 7.61	-	15.53 ± 1.79	43.80 ± 3.48	54.82 ± 5.84	175.78 ± 17.63
August	69.90 ± 6.35	-	19.22 ± 1.62	31.23 ± 0.08	27.86 ± 0.73	148.22 ± 5.06
September	10.51 ± 0.11	5.86 ± 0.05	0.44 ± 0.04	4.45 ± 0.02	4.41 ± 0.10	25.67 ± 0.23
*Felina 32*						
June	53.79 ± 7.79	-	1.32 ± 1.28	51.33 ± 4.74	18.36 ± 0.99	124.79 ± 14.59
July	16.06 ± 1.75	-	8.08 ± 1.04	35.53 ± 1.66	30.55 ± 1.34	90.22 ± 4.32
August	49.72 ± 2.69	-	1.00 ± 1.31	26.91 ± 2.35	40.38 ± 2.67	118.01 ± 6.93
September	75.53 ± 4.85	6.64 ± 0.40	13.71 ± 0.33	31.77 ± 1.99	33.63 ± 1.96	161.28 ± 8.67
*Fedora 17*						
September	18.46 ± 0.77	5.98 ± 0.07	7.05 ± 1.52	42.09 ± 5.59	34.82 ± 4.83	108.40 ± 12.51

## References

[1] EUR-Lex Access to European Union Law. https://eur-lex.europa.eu/legal-content/EN/TXT/?uri=CELEX:01999R1251-20040701.

[2] Ingrao C., Lo Giudice A., Bacenetti J., Tricase C., Dotelli G., Fiala M., Siracusa V., Mbohwa C. (2015). Energy and environmental assessment of industrial hemp for building applications: A review. Renew. Sustain. Energy Rev..

[3] Kreuger E., Prade T., Escobar F., Svensson S.E., Englund J.E., Björnsson L. (2011). Anaerobic digestion of industrial hemp-Effect of harvest time on methane energy yield per hectare. Biomass Bioenergy.

[4] Lühr C., Pecenka R., Budde J., Hoffmann T., Gusovius H.J. (2018). Comparative investigations of fibreboards resulting from selected hemp varieties. Ind. Crop. Prod..

[5] Croxford J.L., Pryce G., Jackson S.J., Ledent C., Giovannoni G., Pertwee R.G., Yamamura T., Baker D. (2008). Cannabinoid-mediated neuroprotection, not immunosuppression, may be more relevant to multiple sclerosis. J. Neuroimmunol..

[6] Appendino G., Gibbons S., Giana A., Pagani A., Grassi G., Stavri M., Smith E., Rahman M.M. (2008). Antibacterial cannabinoids from Cannabis sativa: A structure-activity study. J. Nat. Prod..

[7] Burstein S. (2015). Cannabidiol (CBD) and its analogs: A review of their effects on inflammation. Bioorganic Med. Chem..

[8] Vogl C.R., Mölleken H., Lissek-Wolf G., Surböck A., Kobert J. (2004). Hemp (Cannabis sativa L.) as a resource for green cosmetics: Yield of seed and fatty acid compositions of 20 varieties under the growing conditions of organic farming in Austria. J. Ind. Hemp.

[9] Callaway J.C. (2004). Hempseed as a nutritional resource: An overview. Euphytica.

[10] Stott C.G., Guy G.W. (2004). Cannabinoids for the pharmaceutical industry. Euphytica.

[11] Verma R.S., Padalia R.C., Verma S.K., Chauhan A., Darokar M.P. (2014). The essential oil of “bhang” (Cannabis sativa L.) for non-narcotic applications. Curr. Sci..

[12] Thomas B.F., ElSohly M.A. (2015). The Analytical Chemistry of Cannabis: Quality Assessment, Assurance, and Regulation of Medicinal Marijuana and Cannabinoid Preparations.

[13] Campiglia E., Radicetti E., Mancinelli R. (2017). Plant density and nitrogen fertilization affect agronomic performance of industrial hemp (Cannabis sativa L.) in Mediterranean environment. Ind. Crop. Prod..

[14] Bertoli A., Tozzi S., Pistelli L., Angelini L.G. (2010). Fibre hemp inflorescences: From crop-residues to essential oil production. Ind. Crop. Prod..

[15] Ascrizzi R., Ceccarini L., Tavarini S., Flamini G., Angelini L.G. (2019). Valorisation of hemp inflorescence after seed harvest: Cultivation site and harvest time influence agronomic characteristics and essential oil yield and composition. Ind. Crop. Prod..

[16] Consiglio Regionale del Lazio Interventi per promuovere la coltivazione della canapa (Cannabis Sativa) per scopi produttivi, alimentari ed ambientali e relative filiere. http://www.consiglio.regione.lazio.it/consiglio-regionale/?vw=leggiregionalidettaglio&id=9307&sv=vigente.

[17] Ingallina C., Capitani D., Mannina L., Carradori S., Locatelli M., Di Sotto A., Di Giacomo S., Toniolo C., Pasqua G., Valletta A. (2020). Phytochemical and biological characterization of Italian “sedano bianco di Sperlonga” Protected Geographical Indication celery ecotype: A multimethodological approach. Food Chem..

[18] Sobolev A.P., Mannina L., Capitani D., Sanzò G., Ingallina C., Botta B., Fornarini S., Crestoni M.E., Chiavarino B., Carradori S. (2018). A multi-methodological approach in the study of Italian PDO “Cornetto di Pontecorvo” red sweet pepper. Food Chem..

[19] Sobolev A.P., Thomas F., Donarski J., Ingallina C., Circi S., Cesare Marincola F., Capitani D., Mannina L. (2019). Use of NMR applications to tackle future food fraud issues. Trends Food Sci. Technol..

[20] Sobolev A.P., Circi S., Capitani D., Ingallina C., Mannina L. (2017). Molecular fingerprinting of food authenticity. Curr. Opin. Food Sci..

[21] Happyana N., Kayser O. (2016). Monitoring Metabolite Profiles of Cannabis sativa LTrichomes during Flowering Period Using 1H-NMR-Based Metabolomics and Real-Time PCR. Planta Med..

[22] Flores-Sanchez I.J., Peč J., Fei J., Choi Y.H., Dušek J., Verpoorte R. (2009). Elicitation studies in cell suspension cultures of Cannabis sativa L. J. Biotechnol..

[23] Choi Y.H., Kim H.K., Hazekamp A., Erkelens C., Lefeber A.W.M., Verpoorte R. (2004). Metabolomic differentiation of Cannabis sativa cultivars using 1H-NMR spectroscopy and principal component analysis. J. Nat. Prod..

[24] Nagy D.U., Cianfaglione K., Maggi F., Sut S., Dall’Acqua S. (2019). Chemical Characterization of Leaves, Male and Female Flowers from Spontaneous Cannabis (Cannabis sativa L.) Growing in Hungary. Chem. Biodivers..

[25] Mazzoccanti G., Ismail O.H., D’Acquarica I., Villani C., Manzo C., Wilcox M., Cavazzini A., Gasparrini F. (2017). Cannabis through the looking glass: Chemo- and enantio-selective separation of phytocannabinoids by enantioselective ultra high performance supercritical fluid chromatography. Chem. Commun..

[26] Glória M.B.A., Tavares-Neto J., Labanca R.A., Carvalho M.S. (2005). Influence of cultivar and germination on bioactive amines in soybeans (Glycine max L. Merril). J. Agric. Food Chem..

[27] Sikora V., Berenji J., Latković D. (2011). Influence of agroclimatic conditions on content of main cannabinoids in industrial hemp (Cannabis sativa L.). Genetika.

[28] Brighenti V., Pellati F., Steinbach M., Maran D., Benvenuti S. (2017). Development of a new extraction technique and HPLC method for the analysis of non-psychoactive cannabinoids in fibre-type Cannabis sativa L. (hemp). J. Pharm. Biomed. Anal..

[29] Mandrioli M., Tura M., Scotti S., Toschi T.G. (2019). Fast Detection of 10 Cannabinoids by RP-HPLC-UV Method in Cannabis sativa L. Molecules.

[30] Pollastro F., Minassi A., Fresu L.G. (2017). Cannabis Phenolics and their Bioactivities. Curr. Med. Chem..

[31] Flores-Sanchez I.J., Verpoorte R. (2008). Secondary metabolism in cannabis. Phytochem. Rev..

[32] Zengin G., Menghini L., Sotto A.D., Mancinelli R., Sisto F., Carradori S., Cesa S., Fraschetti C., Filippi A., Angiolella L. (2018). Chromatographic analyses, in vitro biological activities, and cytotoxicity of cannabis sativa l. Essential oil: A multidisciplinary study. Molecules.

[33] Ferrante C., Recinella L., Ronci M., Menghini L., Brunetti L., Chiavaroli A., Leone S., Di Iorio L., Carradori S., Tirillini B. (2019). Multiple pharmacognostic characterization on hemp commercial cultivars: Focus on inflorescence water extract activity. Food Chem. Toxicol..

[34] Martínez-Villaluenga C., Gulewicz P., Pérez A., Frías J., Vidal-Valverde C. (2006). Influence of lupin (Lupinus luteus L. cv. 4492 and Lupinus angustifolius L. var. zapaton) and fenugreek (Trigonella foenum-graecum L.) germination on microbial population and biogenic amines. J. Agric. Food Chem..

[35] Bartkiene E., Juodeikiene G., Vidmantiene D. (2012). Nutritional quality of fermented defatted soya and flaxseed flours and their effect on texture and sensory characteristics of wheat sourdough bread. Int. J. Food Sci. Nutr..

[36] Musarra M., Jirillo R., Rapa M., Vinci G. (2019). Canapa sativa L. and Moringa oleifera as Naturally Functional Beverages: Innovative Trends. Natural Beverages.

[37] Sánchez-Pérez S., Comas-Basté O., Rabell-González J., Veciana-Nogués M.T., Latorre-Moratalla M.L., Vidal-Carou M.C. (2018). Biogenic amines in plant-origin foods: Are they frequently underestimated in low-histamine diets?. Foods.

[38] Chandra S., Lata H., ElSohly M.A., Walker L.A., Potter D. (2017). Cannabis cultivation: Methodological issues for obtaining medical-grade product. Epilepsy Behav..

[39] BLIGH E.G., DYER W.J. (1959). A rapid method of total lipid extraction and purification. Can. J. Biochem. Physiol..

[40] Capitani D., Mannina L., Proietti N., Sobolev A.P., Tomassini A., Miccheli A., Di Cocco M.E., Capuani G., De Salvador R., Delfini M. (2010). Monitoring of metabolic profiling and water status of Hayward kiwifruits by nuclear magnetic resonance. Talanta.

